# Tongue Cancer in a Young Male

**DOI:** 10.7759/cureus.17702

**Published:** 2021-09-03

**Authors:** Gyanendra Bagale, Sandip R Pradhan, Archana Basnet

**Affiliations:** 1 Otorhinolaryngology, Patan Academy of Health Sciences, Kathmandu, NPL; 2 Internal Medicine, Lumbini Medical College, Palpa, NPL; 3 Medicine, De La Salle Medical and Health Sciences Institute, Cavite, PHL

**Keywords:** tongue cancer, oral cancer, squamous cell carcinoma, young adults, betel nuts

## Abstract

Squamous cell carcinoma is a common cancer of the oral cavity. The median age of presentation is 60 years and it rarely occurs in patient aged less than 30 years. We present a case of a 29-year-old male who had ulceroproliferative growth at the left lateral aspect of the tongue. He had a risk factor of tobacco limed betel nut chewing from the age of 14. After detailed investigations, squamous cell carcinoma was confirmed with a staging of cT4a N0 M0. He underwent surgery along with radiotherapy afterward.

## Introduction

Globally, oral cancers accounted for 300,373 new cancer cases and 145,353 cancer deaths in 2012. More than half of oral cancers globally occur in Asia, where it was estimated that 168,850 new cases were diagnosed in this geographical region alone [1]. The responsible etiological factors are smoking, tobacco chewing, alcohol, and sharp jagged teeth causing chronic irritation [2]. Besides viruses like the human papilloma virus, herpes simplex virus and inherited syndromes, (e.g., Plummer‐Vinson syndrome) may also play a role in the carcinogenic process [3]. The premalignant lesions of the tongue are erythroplakia, leukoplakia, lichen planus, and atrophic glossitis [4].

The incidence of oral cancer ranged from 1.6 to 8.6/100,000 per annum, with similar rates in males and females in South East Asia [5]. Those rates are on the increase among the population less than 35 years. Only approximately 2% of patients are diagnosed before the age of 35 [6]. Studies have reported that risk factors of smoking and drinking, considered significant etiological agents in older patients, were also present to varying degrees in younger people. However, there is disagreement about whether these factors may be contributory in young people due to the relatively short time frame of exposure.

Patients in the younger age group have a more aggressive disease with a higher incidence of local recurrence or regional lymph node involvement after treatment and a higher mortality rate than older patients [7].

## Case presentation

A 29-year-old male presented to the hospital with complaints of pain and ulcer on the left side of the tongue for five months. The pain aggravated while speaking and chewing food. In addition, he complained of occasional bleeding from the swelling, along with left earache. However, he did not complain of headaches, changes in speech, difficulty in opening his mouth, and swelling of the neck. He was chronic tobacco and limed betel nut chewer for the last 12 years.

**Figure 1:**
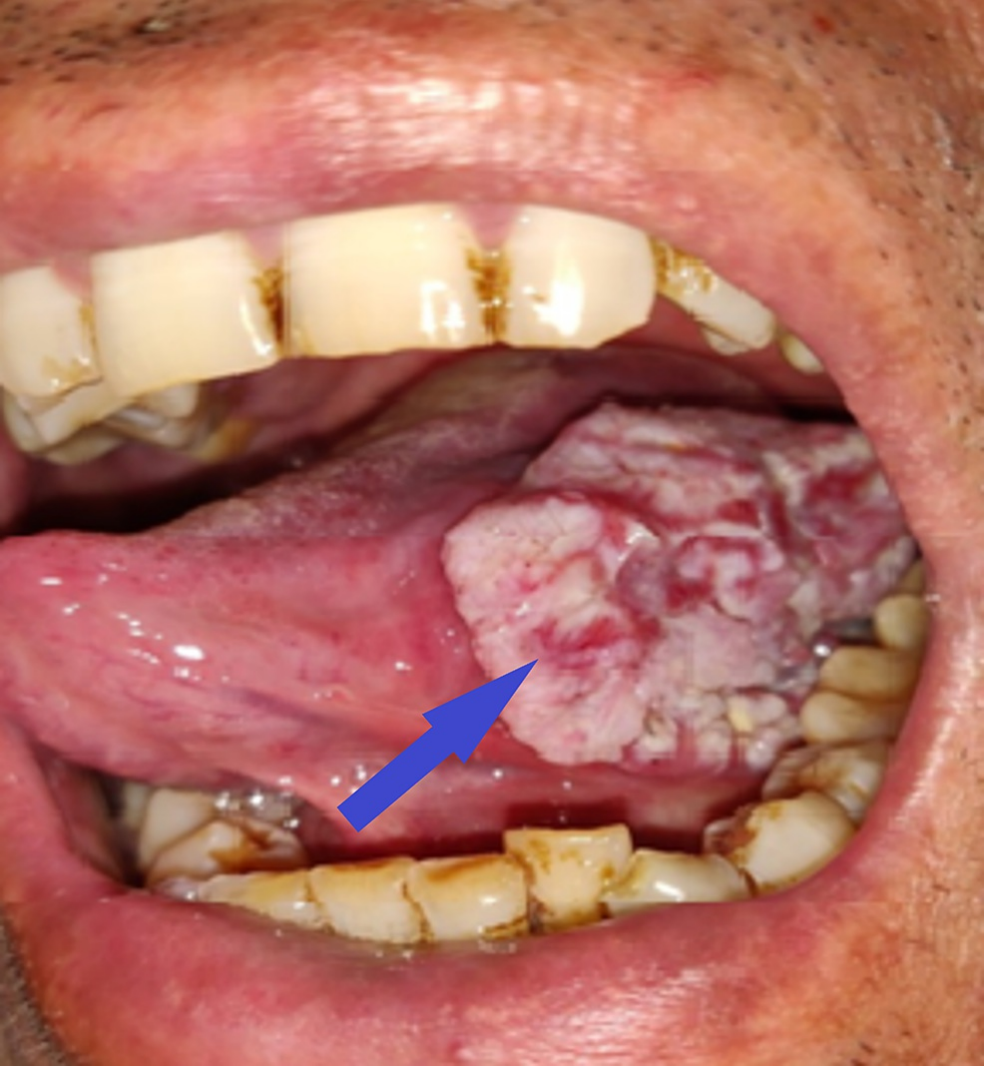
Ulcero-proliferative lesion in the left lateral border of the tongue with generalized attrition and stains on the teeth (blue arrow)

He was well-nourished with a body mass index (BMI) of 23 kg/m2. On oral cavity examination, there was generalized attrition and stains on the teeth with ulcero-proliferative growth in the left lateral border of the tongue (Figure 1). The lesion measured 5 × 3 cm with mixed white and red in color; it had an exophytic and fungating surface, and ill-defined margins. It was located at about 3 cm from the tip of the tongue and 2 cm from the midline. The left anterior tonsillar pillar was about 1 cm from the posterior margin of the ulcer. In addition, there was the involvement of the left gingivo-lingual sulcus and floor of the mouth posteriorly. It was firm in consistency on palpation, and 1.5 cm of the surrounding areas from the ulcer were indurated. It was tender and fixed to the underlying tissues. It bled on the removal of the slough covering the floor. However, movement of the tongue was not restricted, and retro-molar trigone was also normal.

On neck examination, there were enlarged cervical lymph nodes at left level Ib and IIa, the largest measuring about 2 x 2 cm in size. It was non-tender, firm, and mobile. After this, a punch biopsy of ulcero-proliferative growth was performed. The result were positive for squamous cell carcinoma (moderately differentiated).

**Figure 2:**
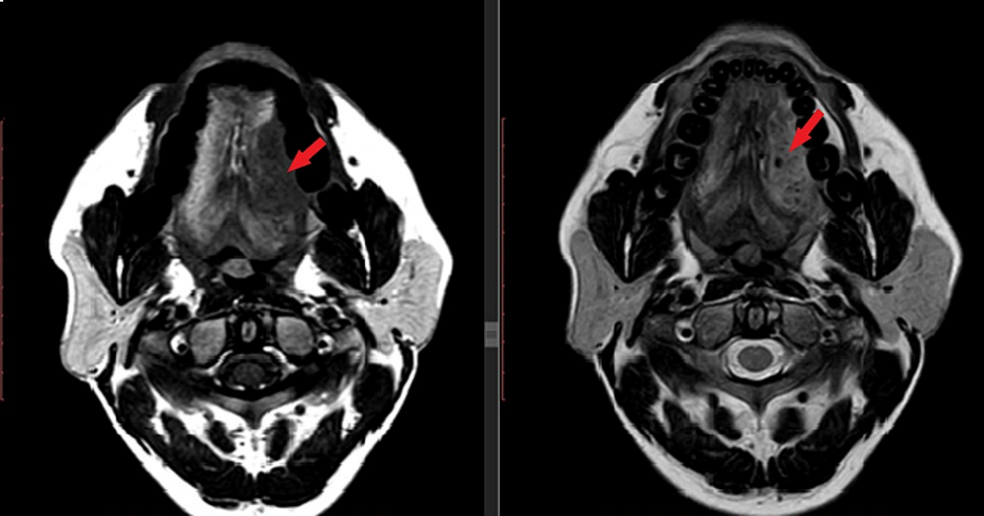
Plain MRI of the lesion at the left lateral border of the tongue showing T1 low signal and T2 high signal abnormality (red arrow)

**Figure 3:**
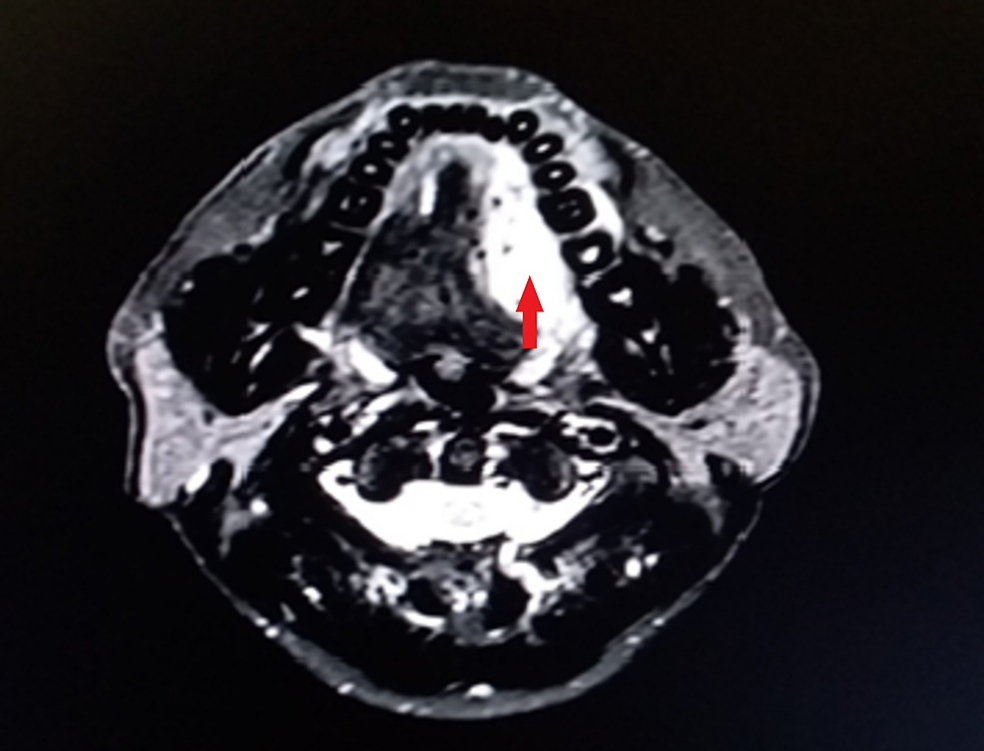
Contrast-enhanced T2 MRI of the lesion showing hyperintensity (red arrow) at the left lateral border

**Figure 4:**
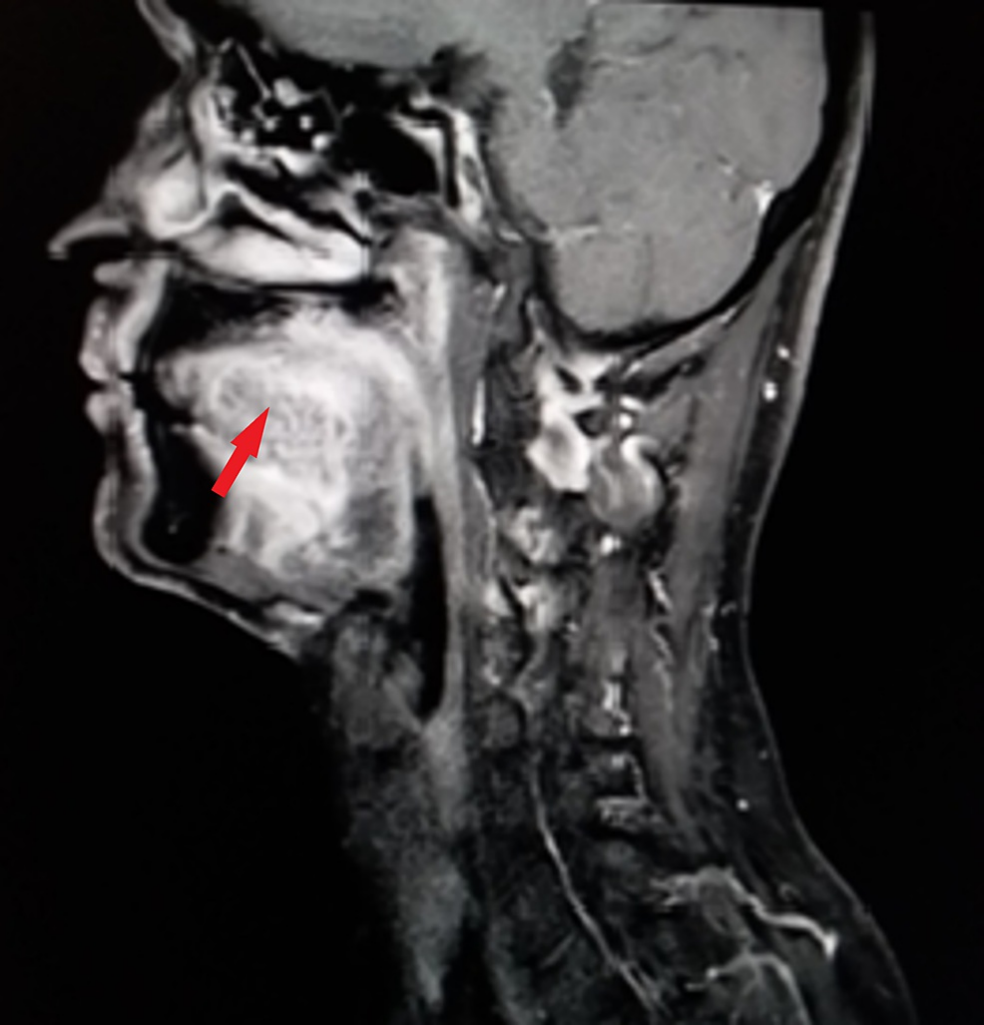
A sagittal section contrast-enhanced MRI showing hyperintensity of the lesion (red arrow)

Subsequently, contrast-enhanced MRI from skull base to superior mediastinum was performed (Figures 2-4). It showed enhancing lesion of size 4.2 x 2.2 x 4.3 cm in size at the left lateral aspect of the tongue, abutting the left palatine tonsil posteriorly with involvement of genioglossus and the sublingual region, and enhancement in the adjacent left mandible.

Fine needle aspiration cytology (FNAC) from the left level IIa lymph node showed reactive lymphadenitis. Hence, the clinical diagnosis of carcinoma tongue was confirmed. The staging was carcinoma left lateral border of tongue cT4a N2b M0.

**Figure 5:**
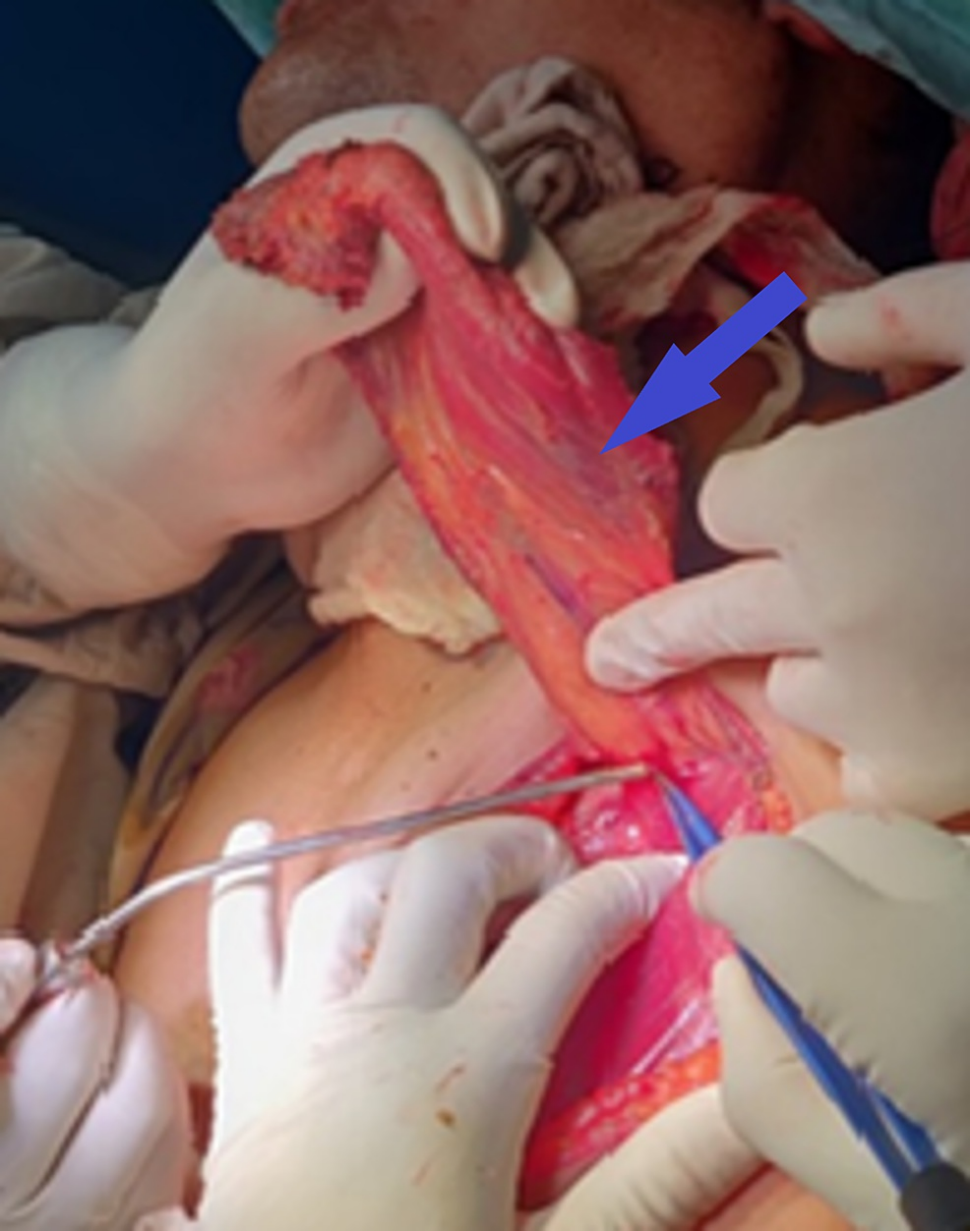
Pectoralis major myocutaneous flap (blue arrow)

**Figure 6:**
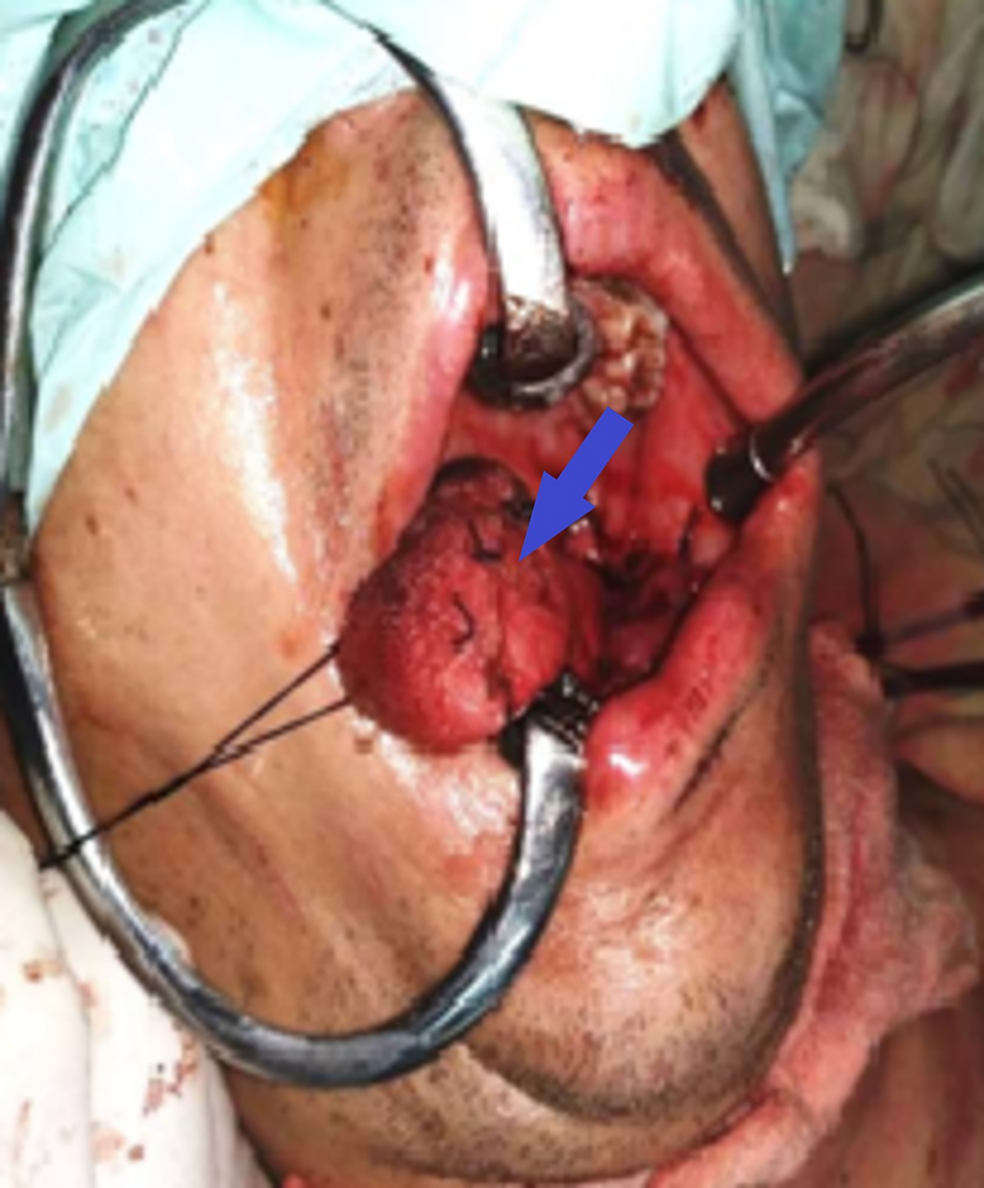
Oral cavity and tongue after hemiglossectomy and repair (blue arrow)

He underwent left hemiglossectomy with marginal mandibulectomy with extended supraomohyoid neck dissection with pectoralis major myocutaneous flap with tracheostomy (Figures 5-6).

After surgery, the final histopathological report confirmed squamous cell carcinoma measuring 4.2 cm at its largest dimension with a depth of invasion of 7.8 mm. All the surgical margins were not involved by the tumor. The closest surgical margin was 0.6 cm from the base. The bone was not involved. The lymphovascular and perineural invasion was not observed. The lymph nodes from levels 1A, 1B, 2A, 2B, 3, and 4 were not involved. The final pathological staging was pT3 N0. The patient underwent radiotherapy on the 6th week after surgery. He underwent radiation therapy and received 60 grays of radiation for five days a week for one month.

**Figure 7:**
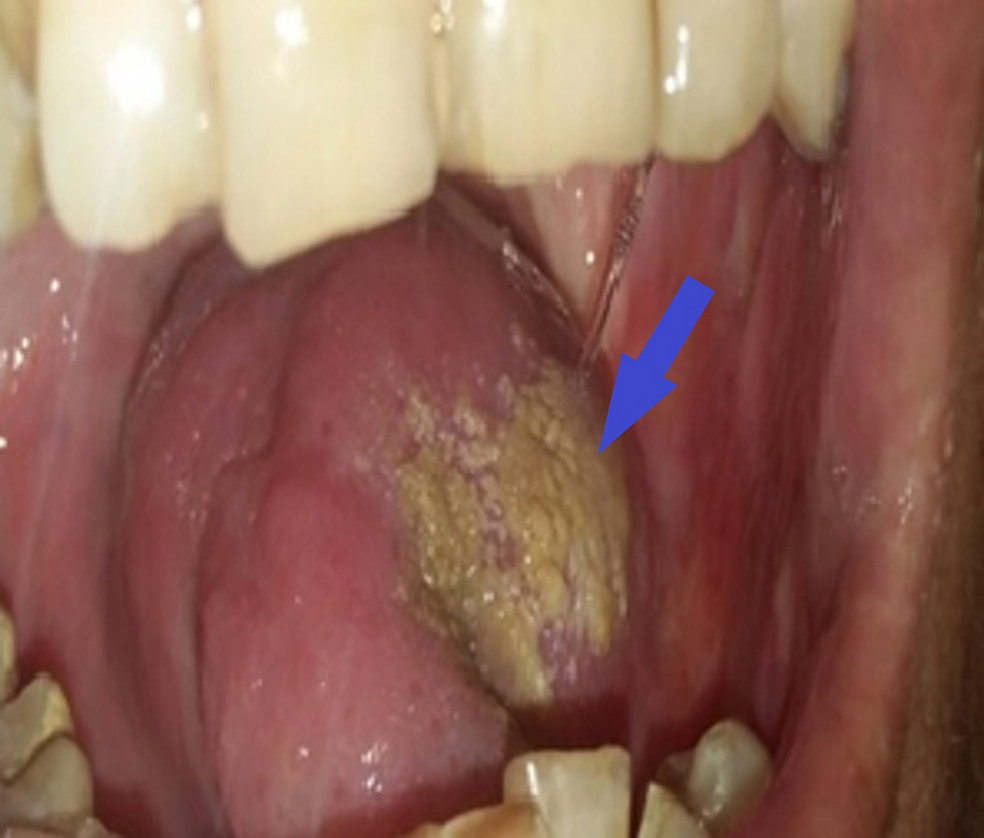
Post-surgery status of the tongue during the follow-up (blue arrow)

Currently, he is asymptomatic with normal clinical findings after two years of treatment (Figure 7). He is under our regular follow-up.

## Discussion

The median age of diagnosis of tongue cancer is 60 years. Only about 2% are present below the age of 35 years [4]. Squamous cell carcinoma is the most common histological variant. It is more common in males than females, with a ratio of 2: 1. The incidence is higher in Asia. It may be due to different social habits such as chewing betel nuts mixed with lime and the habit of reverse smoking. Most people start these habits from a very young age. Therefore, it might be the cause of the growing incidence of young age cancer in these countries.

Our patient was a male with regular limed betel nuts chewing and tobacco habit since age 14. Radiographic assessment with CT scan and MRI is of great importance in cancer of the tongue. They help identify the greatest dimension of a tumor, depth of invasion, involvement of extrinsic muscle of tongue, maxillary sinus, masticator space, cortical bone, and pterygoid plates. It also helps to identify the nodal number, size, location, contour, and necrosis. Ultimately it helps in staging tumors.

This should be followed by a biopsy. Our patient had a tumor of size 4.2 x 2.2 x 4.3 cm at the left lateral aspect of the tongue with involvement of genioglossus, sublingual region, and enhancement in the adjacent left mandible. The punch biopsy showed squamous cell carcinoma. However, the FNAC from lymph was negative for malignancy. Hence, the staging was cT4a N0 M0. However, treatment depends on the staging of the tumor. For T4, tumor treatment of choice is wide local excision with supra-omohyoid neck dissection followed by radiotherapy.

Besides, our patient underwent a marginal mandibulectomy to get a clear surgical margin. Ideally, for closure of defects at the floor of the mouth, a free flap is preferred. However, our patient underwent pectoralis major myocutaneous flap due to low resource setting.

## Conclusions

Squamous cell carcinoma of the tongue which is highly prevalent in old age people, is increasing in trend among the younger generation due to early exposure to risk factors like limed betel nuts chewing and tobacco consumption which are common in developing countries.
